# Overexpression of DAZL, STRA8, and BOULE Genes and Treatment With BMP4 or Retinoic Acid Modulate the Expression of MSC Overexpressing Germ Cell Genes

**DOI:** 10.3389/fvets.2021.667547

**Published:** 2021-05-25

**Authors:** Paloma Cordero, Alejandra Guerrero-Moncayo, Monica De los Reyes, Manuel Varas-Godoy, Jahaira Cortez, Cristian G. Torres, Victor H. Parraguez, Oscar A. Peralta

**Affiliations:** ^1^Department of Animal Production Sciences, Faculty of Veterinary and Animal Sciences, University of Chile, Santiago, Chile; ^2^Centro de Biología Celular y Biomedicina (CEBICEM), Facultad de Medicina y Ciencia, Universidad San Sebastián, Santiago, Chile; ^3^Department of Clinical Sciences, Faculty of Veterinary and Animal Sciences, University of Chile, Santiago, Chile; ^4^Department of Biological Sciences, Veterinary and Animal Sciences, University of Chile, Santiago, Chile

**Keywords:** mesenchymal stem cells, bovine fetus, germ cell differentiation, polycistronic vector, bone morphogenetic protein 4, retinoic acid

## Abstract

*In vitro* gamete derivation from stem cells has potential applications in animal reproduction as an alternative method for the dissemination of elite animal genetics, production of transgenic animals, and conservation of endangered species. Mesenchymal stem cells (MSCs) may be suitable candidates for *in vitro* gamete derivation considering their differentiative capacity and their potential for cell therapy. Due to its relevance in gametogenesis, it has been reported that retinoic acid (RA) and bone morphogenetic protein (BMP) 4 are able to upregulate the expression of specific markers associated to the early stages of germ cell (GCs) differentiation in bovine fetal MSCs (bfMSCs). In the present study, we used polycistronic vectors containing combinations of GC genes DAZL, STRA8, and BOULE followed by exposure to BMP4 or RA to induce GC differentiation of bovine fetal adipose tissue-derived MSC (AT-MSCs). Cells samples at Day 14 were analyzed according to the expression of pluripotent genes NANOG and OCT4 and GC genes DAZL, STRA8, BOULE, PIWI, c-KIT, and FRAGILIS using Q-PCR. Fetal and adult testis and AT-MSCs samples were also analyzed for the expression of DAZL, STRA8, and NANOG using immunofluorescence. Increased gene expression levels in the adult testis and cell-specific distribution of DAZL, STRA8, and NANOG in the fetal testis suggest that these markers are important components of the regulatory network that control the *in vivo* differentiation of bovine GCs. Overexpression of DAZL and STRA8 in bi-cistronic and DAZL, STRA8, and BOULE in tri-cistronic vectors resulted in the upregulation of OCT4, NANOG, and PIWIL2 in bovine fetal AT-MSCs. While BMP4 repressed NANOG expression, this treatment increased DAZL and c-KIT and activated FRAGILIS expression in bovine fetal AT-MSCs. Treatment with RA for 14 days increased the expression of DAZL and FRAGILIS and maintained the mRNA levels of STRA8 in bovine fetal AT-MSCs transfected with bi-cistronic and tri-cistronic vectors. Moreover, RA treatment repressed the expression of OCT4 and NANOG in these cells. Thus, overexpression of DAZL, STRA8, and BOULE induced the upregulation of the pluripotent markers and PIWIL2 in transfected bovine fetal AT-MSCs. The partial activation of GC gene expression by BMP4 and RA suggests that both factors possess common targets but induce different gene expression effects during GC differentiation in overexpressing bovine fetal AT-MSCs.

## Introduction

*In vitro* gamete derivation from stem cells has a promising potential role for the treatment of male infertility in human reproductive medicine (1, 2). This technology may also expand its potential applications to animal reproduction as an alternative method for the dissemination of elite animal genetics, production of transgenic animals, and conservation of endangered species. In the case of a cattle, large-scale culture of *in vitro*-produced germ cells (GCs) would allow a continuous supply of elite animal genetics for transplantation into the seminiferous tubules of recipient animals in a donor-derived spermatogenesis (3).

Mesenchymal stem cells isolated from bovine fetal tissues (bfMSCs) posses the intrinsic capacity for differentiation into osteogenic, chondrogenic, and adipogenic lineages (4, 5). Plasticity of bfMSCs is not limited to mesodermal derivatives since these cells were also able to differentiate into neuroectodermal and endodermal cell types (6). Recently, it has been reported that bfMSCs exposed to bioactive factors, including retinoic acid (RA) and bone morphogenetic protein (BMP) 4 or cocultured with Sertoli cells, are able to upregulate the expression of specific markers associated to the early stages of GC differentiation (7, 8). In addition, bfMSCs can be easily isolated and expanded under *in vitro* conditions to obtain a sufficient number for transplantation. Moreover, bfMSCs express low levels of immunogenic markers (MHC-I, MHC-II, CD80, and CD86), display immune suppressive capacity (9), and possess a reduced intrinsic teratogenic potential (10). Overall, these features suggest that bfMSCs may be used for *in vitro* GC production and allogenic transplantation into the testis of recipient animals.

In domestic animals, male GCs are derived from a population of primordial germ cells (PGCs) which originated in the proximal epiblast (11). PGC specification is induced by extrinsic factors secreted by extraembryonic ectoderm, including BMP4 that activates the expression of GC-specific marker FRAGILIS in the proximal epiblast, marking the first step in germ-line commitment (12). In mice, the subset of presumptive GCs positive for FRAGILIS migrates to the extraembryonic mesoderm and suppresses somatic cell gene expression, promoting the expression of pluripotent genes octamer-binding transforming factor 4 (OCT4) and NANOG (13). During migration through the hindgut to the genital ridge developing into the future testis, PGCs start expressing deleted in azoospermia like (DAZL), which plays essential roles in the development of PGCs and in the differentiation and maturation of GCs (14). After migration, mice PGCs enter a mitotic arrest and are reactivated after birth to initiate spermatogenesis. It has been reported that stimulated by RA8 (STRA8) is an essential factor involved in the decision of meiotic entry of postmigratory PGCs, in a process highly regulated by RA (15, 16). Thus, upregulation of STRA8 suggests that the GCs are ready for meiotic pairing and recombination of homologous chromosomes (17).

The relevance of DAZL has been demonstrated in knockout mice, where embryos display a reduced expression of GC-specific genes and postnatal males present an impairment in the progression from A to A1 spermatogonia and meiotic arrest resulting in azoospermia and sterility (18, 19). DAZL is expressed throughout most of the life of GCs and is required for the development of PGCs and for the differentiation and maturation of GCs (14). In addition, BOULE (also called BOLL), a member *via* RNA-binding protein of the deleted in azoospermia (DAZ) gene family, complements the functions of DAZL and has been reported to be important for meiotic division and spermatogenesis (20). All evidence indicates that DAZL and BOULE have synergistic effects and are fundamental for spermatogenesis (21). Recent studies indicate that the overexpression of STRA8, BOULE, and DAZL promotes the transdifferentiation of goat BM-MSCs to early GC–like cells *in vitro* (22, 23); however, this approach has not been attempted in bfMSCs or bovine adult MSCs. Thus, considering that the co-expression of multiple genes is an interesting strategy for promoting cell differentiation, and the crucial effects of BMP4 and RA on GC development, in the present study, we used polycistronic vectors containing combinations of DAZL, STRA8, and BOULE genes followed by exposure to BMP4 or RA to induce GC differentiation of bovine fetal adipose tissue-derived MSC (AT-MSCs).

## Materials and Methods

### Study Design

**Figure 1:**
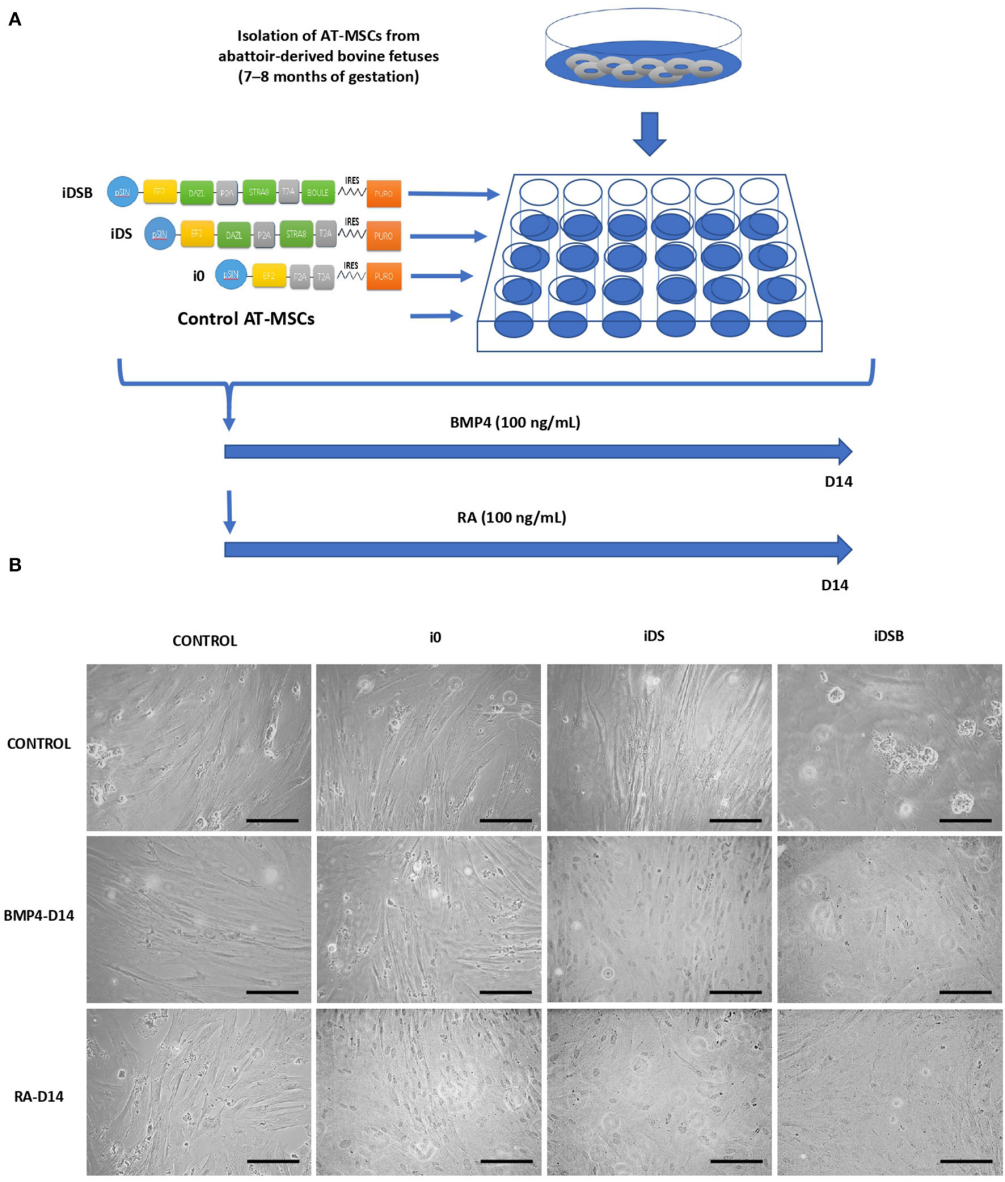
Experimental design for transfection of polycistronic vectors expressing DAZL, STRA8, and BOULE in bovine fetal AT-MSCs and subsequent treatments with BMP4 and RA. **(A)** AT-MSCs were transfected with tri-cistronic vectors containing a combination of DAZL, STRA8, and BOULE genes (iDSB), bi-cistronic vector carrying DAZL and STRA8 genes (iDS), or empty vector (i0). Control treatment were non-transfected AT-MSCs. Thereafter, transfected and control AT-MSCs were treated with 100 ng/mL of BMP4 or 0.1 μM of RA for 14 days. **(B)** AT-MSCs expressing i0, iDS, and iDSB had a similar fibroblast-like morphology and culture organization compared to control AT-MSCs. Control AT-MSCs transfected with iDSB formed scattered cell aggregates in cell cultures at Day 14. Furthermore, transfected AT-MSCs with iDS and iDSB vectors and treated with BMP4 or RA displayed similar a culture organization compared to the controls with a confluency and fibroblast-like morphology. Scale bars: 500 mm.

All procedures have been approved by the Bioethical Committee of the National Commission for Scientific and Technology Research from Chile (Fondecyt). The developmental gene expression of pluripotent OCT4 and NANOG and GC markers DAZL, STRA8, BOULE, PIWI, c-KIT, and FRAGILIS was evaluated in a bovine testis at gestational months 6 (M6) and 9 (M9) and at 1 year (1Y) old. Testis samples were collected from bovine fetuses (*n* = 5 for each stage) and adults (*n* = 5), and protein expression of DAZL, STRA8, and NANOG were analyzed using immunofluorescence. Furthermore, the effects of the ectopic expression of combinations of DAZL, STRA8, and BOULE using bi-cistronic iDS or tri-cistronic iDSB vectors were evaluated on the potential GC differentiation of bovine fetal AT-MSCs (Figure 1A). Control treatments were AT-MSCs transfected with empty i0 vector and non-transfected AT-MSCs. Tissue samples of AT were isolated from bovine fetuses (*n* = 9) and were pooled separately (each pool from three fetuses). Each pool represented a biological replicate and analyses and experiments were performed in sextuplicate. Pooling tissue samples was performed in an effort to reduce individual biological variation. Furthermore, transfected and control AT-MSCs were treated with 100 ng/mL of BMP4 or 0.1 μM of RA for 14 days (7, 8). Cells samples at Day 14 were analyzed for the expression of pluripotent genes NANOG and OCT4 and GC genes DAZL, STRA8, BOULE, PIWIL2, c-KIT, and FRAGILIS using Q-PCR. Samples were also analyzed for the protein expression of DAZL, STRA8, and NANOG using immunofluorescence.

### Isolation and Culture of Bovine Fetal AT-MSCs

AT-MSCs were isolated following a previously reported protocol that ensured the establishment of MSC cultures that fulfill the minimal criteria for the definition of MSCs (4, 5). AT-MSCs were harvested from the omentum of male bovine fetuses (*n* = 9; 7–8 months of gestation) collected at a local abattoir. Approximately 10 g of AT was isolated under aseptic conditions and deposited in a phosphate-buffered saline (PBS; Hyclone, Thermo Fisher Scientific, UT, USA) supplemented with 100 IU/mL of penicillin, 100 μg/mL of streptomycin, and 2.5 μg/mL of amphotericin B (Hyclone). AT samples from three fetuses were pooled and washed twice with PBS and twice with Dulbecco's Modified Eagle Medium (DMEM; Hyclone). Then, AT was digested in 0.5% collagenase I (Sigma-Aldrich, St. Louis MO, USA) (1 mL/g of AT) for 45 min. Collagenase I activity was neutralized with DMEM supplemented with 10% fetal bovine serum (FBS; Gibco, Waltham, MA, USA), 100 IU/mL of penicillin, 100 μg/mL of streptomycin, and 2.5 μg/mL of amphotericin B (expansion medium). The disrupted tissue was filtered through 40 μm pores and subsequently centrifuged at 400 x g for 5 min. The cell pellet was washed once in DMEM, suspended in an expansion medium, and plated. Following the determination of cell viability, cells were used to initiate the experiments.

### Synthesis of Bovine Polycistronic Expression Vectors

Expression vector pSIN carrying MF2 promoter, cleaving sequences P2A and T2A, puromycin gene, and integrated bovine genes DAZL, STRA8, and BOULE (pSIN-EF2-DAZL-P2A-STRA8-T2A-BOULE-PURO) (iDSB) or DAZL and STRA8 (pSIN-EF2-DAZL-P2A-STRA8-PURO) (iDS), and empty plasmid (pSIN-EF2-P2A-T2A-PURO) (i0) were purchased from GenScript Biotech Corp (Hong Kong, China). Plasmid DNA was extracted from paper by dilution in 300 μL of nuclease-free water and placed in a water bath at 50°C for 5 min. In order to replicate the plasmid DNA, competent Stbl3 cells (Thermo Fisher Scientific) from a bacterial strain were used. An aliquot of 50 μL of the Stbl3 cell-suspension was mixed with 10–100 ng of each DNA plasmid. After several incubation cycles at different temperatures (30 min on ice, 45 s at 42°C, and 1 min on ice), 250 μL of warm Super Optimal Broth (SOC) medium (Sigma-Aldrich) was added and the suspension was incubated for 60 min at 37°C with shaking at 7 g. Subsequently, DNA plasmids were centrifuged for 3 min at 1,000 g and 100 μL of pellet was removed and seeded with a rake in plates with the Lysogeny broth (LB) medium (Sigma-Aldrich). The plates were incubated for a period of 16 h at 37°C under a humid environment. Colonies obtained after incubation were minced and mixed with the LB medium. A fraction of the suspension was cryopreserved as additional stock using 30% glycerol. Agar gel electrophoresis was carried out on the diluted fraction to determine the presence of the genes of interest in the bacteria obtained from the colonies in culture by means of the application of restriction enzymes for the detection of DNA fragments. To extract the plasmids, the bacteria was expanded in 100 uL of LB medium cultures overnight at 37°C. The purification of the plasmid DNAs was carried out using a NucleoBond Xtra Midi kit (Macherey-Nagel, CA, USA), adding to each pellet 8 mL of Midiprep RNAse A kit buffer (Thermo Fisher Scientific), and then passing it through a lysis buffer for 5 min at 25°C. Subsequently, plasmid filters were equilibrated and washed using the EQU buffer and 5 mL of elution buffer was applied for DNA decantation. Plasmid DNA precipitation was performed with the addition of 3.5 mL of isopropanol, which was then removed by adding 2 mL of 70% ethanol by centrifugation at 15,000 g for 5 min at 4°C.

### AT-MSC Transfection and Treatments With BMP4 and RA

Transfection of the polycistronic vectors containing combinations of integrated DAZL, STRA8, and BOULE genes was carried out in cultures of bovine fetal AT-MSCs using lipofectamine 2000 (Invitrogen, Carlsbad, CA, USA). AT-MSCs at a concentration of 25 × 104 cells/mL were seeded in 24-well plates in an expansion medium until reaching a confluence of 70–90%. After 48 h, the medium was collected and replaced by the Opti-MeM medium supplemented with lipofectamine at a concentration of 1.5 ng/uL. Additionally, 1 ng/uL of tri-cistronic (iDSB), bi-cistronic (iDS), or empty vector (i0) was added to the medium. Thereafter, the effects of RA and BMP4 on the AT-MSCs that were previously transfected and non-transfected were analyzed. AT-MSCs were cultured in a DMEM (low glucose) medium supplemented with 10% FBS, 100 IU/mL of penicillin, 100 μg/mL of streptomycin, and 0.25 μg/mL of amphotericin B. RA and BMP4 were supplemented at concentrations of 0.1 μM and 100 ng/mL, respectively (7) to each AT-MSC culture for 14 days (Figure 1A). The culture medium containing RA and BMP4 was replaced every 2 days and cells samples at day 14 were analyzed for pluripotency- (NANOG and OCT4) and GC- (DAZL, STRA8, BOULE, PIWI, c-KIT, and FRAGILIS) specific marker expression using Q-PCR and for DAZL, STRA8, and NANOG using immunofluorescence.

### mRNA Isolation and cDNA Retrotranscription

Approximately 1 × 10^5^ cells were collected from the transfected and control AT-MSCs and immediately fixed in a lysis buffer (Thermo Fisher Scientific). Total RNA was extracted using the GeneJET RNA purification kit (Thermo Fisher Scientific) according to the manufacturer's instructions. Total RNA was eluted in 50 μL of RNAse free water. The concentration and purity of the RNA in each sample was determined using the Qubit RNA assay kit (Life Technologies, Waltham, MA, USA), and genomic DNA was removed using DNase I, RNase-free (Thermo Fisher Scientific). Samples were subjected to reverse transcription using a cDNA synthesis kit (AffinityScript; Agilent Technologies, CA, USA). The reaction protocol consisted of incubation for 5 min at 25°C, 15 min at 42°C, 5 min at 95°C, and hold at 4°C using a TC1000-G gradient thermocycler (SciLogex, Rocky Hill, CT, USA).

### Quantitative Polymerase Chain Reaction

β-ACTIN and GAPDH (glyceraldehyde 3-phosphate dehydrogenase) were selected as housekeeping genes based on previous analyses from our laboratory that demonstrated high stability in the bfMSC cultures (4, 6). Primers were designed using the PrimerExpress software (Applied Biosystems Incorporated, Foster City, CA, USA) (Table 1). Equivalence of amplification efficiencies among all primer-probe sets was confirmed using serial 3-fold dilutions of AT-MSC cDNA. Each PCR reaction (10 μL) contained the following: 2X Brilliant II SYBR Green QPCR master mix (5 μL; Agilent Technologies), target forward primer (200 nM), target reverse primer (200 nM), cDNA synthesis reaction (1 μL), and nuclease-free PCR-grade water to adjust the final volume. The PCR amplification was carried out in an Eco Real-Time PCR System (Illumina Incorporated, San Diego, CA, USA). Thermal cycling conditions were 95°C for 10 min, followed by 40 repetitive cycles at 95°C for 30 s, and 60°C for 1 min. All reactions were performed in triplicate. In each experiment, the amount of gene expression was recorded as CT values that corresponded to the number of cycles where the fluorescence signal can be detected above a threshold value. The CT averages for each biological replicate were calculated and transformed into relative values through the ΔΔCT formula (24).

**Table 1:** Sequence of primers used for Q-PCR analysis.

**Gene**	**Nucleotide sequence (5^**′**^-3^**′**^)**	**Accession number**
Endogenous genes		
GAPDH	Forward CCTTCATTGACCTTCACTACATGG TCTA Reverse TGGAAGATGGTGATGGCCTTTCCATTG	NM_001034034.2
β- ACTIN	Forward CGCACCACTGGCATTGTCAT Reverse TCCAAGGCGACGTAGCAGAG	NM_173979.3
Pluripotency genes		
OCT4	Forward GAAAGAGAAAGCGGACGAG Reverse GTGAAAGGAGACCCAGCAG	NM_174580.2
NANOG	Forward TAAGCACAGGGGGCAAAAG Reverse ATGGCTAAAAGGGGTGGAGG	NM_001025344.1
Germ cell genes		
FRAGILIS	Forward ATCTGCAGCGAGACCTCTGT Reverse CCGATGGACATGATGATGAG	XM_002697323
DAZL	Forward TCC AAG TTC ACC AGT TCA GG Reverse CGT CTG TAT GCT TCT GTC CAC	NM_001081725.1
PIWIL2	Forward TCGTATTGATGATGTGGATTGG Reverse GGGAGCAGCAGGATTTCAC	XM_617223.3
STRA8	Forward TGTGCCCAGGTGTTCATCTC Reverse GGGGACTGTCACCTCATTGG	XM_015463130
BOULE	Forward TGTCACCTGTGCCTTTGAATAACC Reverse TTTCAAAAGTGACGAAGCCATACC	NM_001102115.1
c-KIT	Forward TACCAACCAAGGCAGACAA Reverse CTTTGAGGCAAGGAACGC	XM_027544520.1

### Indirect Immunofluorescence

The protein expressions of DAZL, STRA8, and NANOG were immunodetected in a bovine fetal (M9) testis obtained at a local abattoir. After extraction from the uterus and placental membranes, the crown-rump length of each fetus was measured in order to estimate the fetal age. Testis samples were collected from bovine fetuses and adults and were fixed in 4% paraformaldehyde and embedded in paraffin blocks. Testis samples were then sectioned at 5–7 mm using a microtome, mounted on adhesive coated slides (Newcomer supply; Middleton, Wisconsin) and incubated overnight at 37°C. Mounted tissues were deparaffinized in xylene and rehydrated in serial alcohol solutions. Slides were subjected to an unmasking protocol by autoclaving at 120°C for 30 min in a citrate buffer (0.01 M; pH 6). Slides were then rinsed two times in PBS and blocked in 2% bovine serum albumin (BSA) diluted in PBS (pH 7.4) for 1 h at 4°C. In order to immunodetect DAZL, STRA8, and NANOG in AT-MSCs, cells were grown in sterile glass coverslips and then fixed using cold methanol at −20°C for 20 min. Subsequently, cells were washed and blocked with 2% BSA diluted in PBS (pH 7.4) for 30 min. The markers were immunodetected using rabbit polyclonal anti-DAZL antibodies (Cat # ab34139; Abcam, MA, USA), mouse monoclonal anti-STRA8 (Cat# sc517364; Santa Cruz Biotechnology, CA, USA), or mouse monoclonal anti-NANOG (Cat# sc-293121; Santa Cruz), diluted in PBS plus 2% BSA (1:100), and incubated overnight at 4°C. After three washings with 2% BSA in PBS, slides and coverslips were incubated with goat anti-rabbit IgG conjugated with FITC antibodies (Cat# ab97050; Abcam, USA) or donkey anti-mouse Alexa fluor IgG (Cat# A21202; Thermo Fisher Scientific) diluted in BSA 2% for 1 h (1:1,000). Finally, slides and coverslips were washed and mounted in DAPI Vectashield mounting media (Cat # H-1200, CA, USA). The samples were observed and photographed using an epifluorescence microscope and a spectral confocal microscope (Nikon, Japan) with camera connected to a computer.

### Data Analysis

Gene expression data were statistically analyzed using the Infostat software (Cordoba, Argentina). Descriptive column statistics of each data set were performed, including the Shapiro–Wilk normality test. Non-Gaussian distribution of data sets were tested for significant differences using the Kruskal–Wallis test in combination with the Dunn's multiple comparison post-test. The significance level was −0.05.

## Results

### Analysis of the mRNA Levels of Pluripotency and Germ Cell Markers and Immunolocalization of DAZL, STRA8, and NANOG in Bovine Fetal Testis

**Figure 2:**
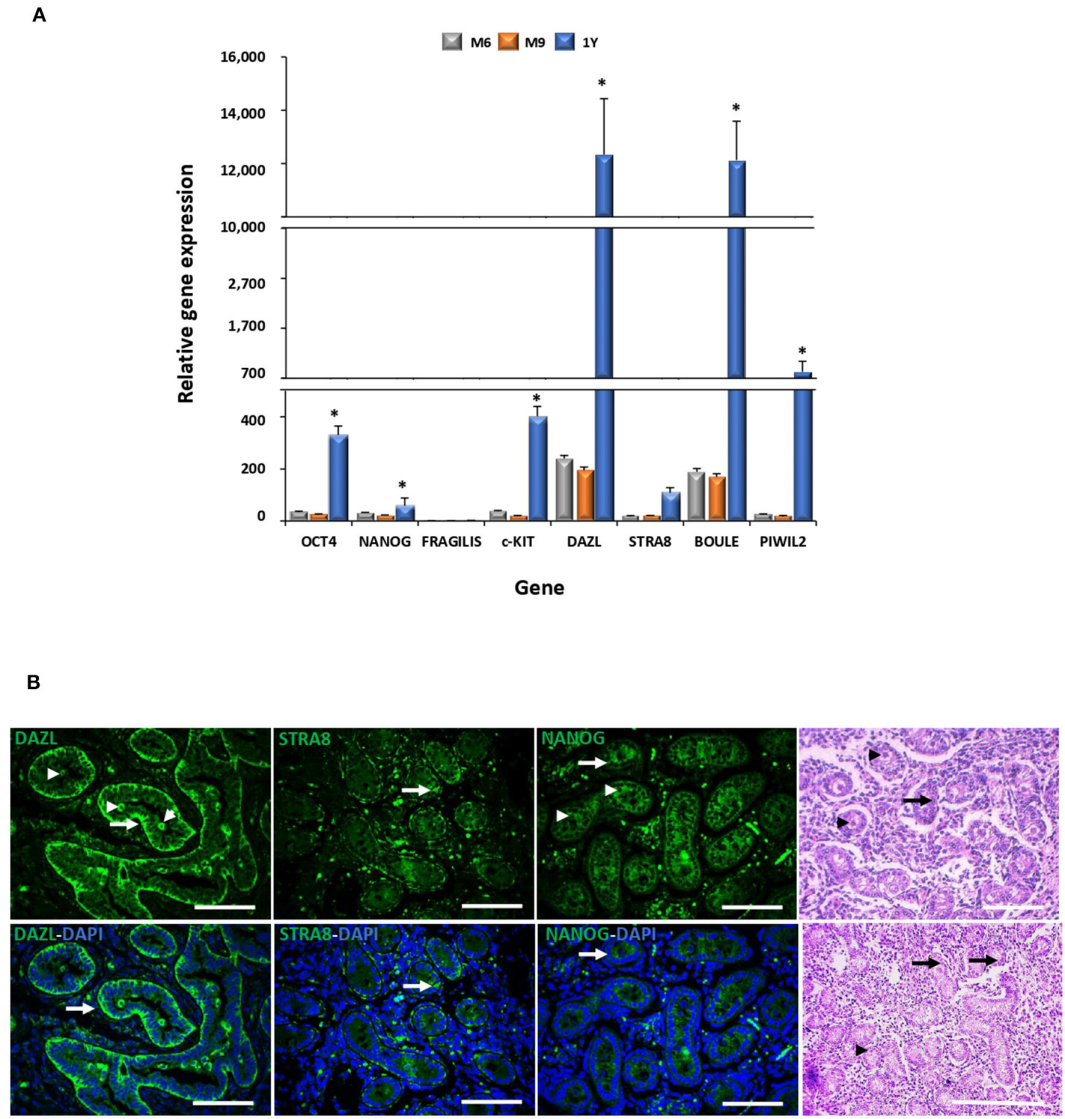
Gene expression analyses of pluripotency and GC markers during bovine testis development and immunodetection of DAZL, STRA8, and BOULE in bovine fetal testis. **(A)** Levels of mRNA of pluripotent OCT4 and NANOG and GC genes c-KIT, DAZL, STRA8, BOULE, and PIWIL2 were higher (*P* < 0.05) in adult compared to fetal testis. **(B)** Sertoli (arrow) and pre-spermatogonial (arrowhead) cells are indicated in epifluorescence and H&E staining of bovine fetal testis (M9). An intense immunosignal associated to DAZL located in Sertoli and pre-spermatogonial cells was detected in the fetal testis. STRA8 immunosignal was observed in the spotted areas mainly in the basal compartment of the seminiferous tubules. NANOG immunosignal was observed across the spermatogenic epithelium with high intensity in Sertoli and pre-spermatogonial cells. (*) indicates significant differences (*P* < 0.05) for gene expression levels between the developmental stages for each gene. Scale bars: 200 μm.

Levels of mRNA of pluripotent genes OCT4 and NANOG and GC genes c-KIT, DAZL, STRA8, BOULE, and PIWIL2 were higher (*P* < 0.05) in the adult compared to the fetal testis (Figure 2A). No significant differences in the mRNA levels of these genes were detected in testis between fetal stages. The expression of DAZL, STRA8, and NANOG was thereafter evaluated during fetal testicular tissue in order to determine its expression in association with gonocytes, pre-spermatogonia, and sertoli cells, which represent the initial stage of germ cell differentiation and a stage more similar to MSC differentiation. An intense immunosignal associated to DAZL probably located in pre-spermatogonial cells was detected in the peripheral areas of seminiferous tubules (Figure 2B) in a 9-month fetal testis. STRA8 immunosignal was observed in the spotted areas mainly in the basal compartment of the seminiferous tubules. Furthermore, we immunodetected NANOG across the spermatogenic epithelium with a high intensity in the Sertoli and pre-spermatogonial cells.

### Morphological Characterization of Bovine Fetal Mesenchymal Stem Cells Transfected With Polycistronic Vectors Carrying Combinations of DAZL, STRA8, and BOULE Genes and Treated With Bone Morphogenetic Factor 4 or Retinoic Acid

Transfected AT-MSCs had a similar fibroblast-like morphology and culture organization compared to the control AT-MSCs (Figure 1B). AT-MSCs transfected with tri-cistronic iDSB formed scattered cell aggregates in cell cultures. Furthermore, the transfected AT-MSCs with bi-cistronic and tri-cistronic vectors and treated with BMP4 or RA displayed a similar culture organization compared to the controls with confluency and fibroblast-like morphology at Day 14.

### Analysis of the mRNA Levels and Immunolocalization of Pluripotency and Germ Cell-Specific Markers in Bovine Fetal Mesenchymal Stem Cells Transfected With Polycistronic Vectors Carrying Combinations of DAZL, STRA8, and BOULE Genes and Treated With Bone Morphogenetic Factor 4 or Retinoic Acid

**Figure 3:**
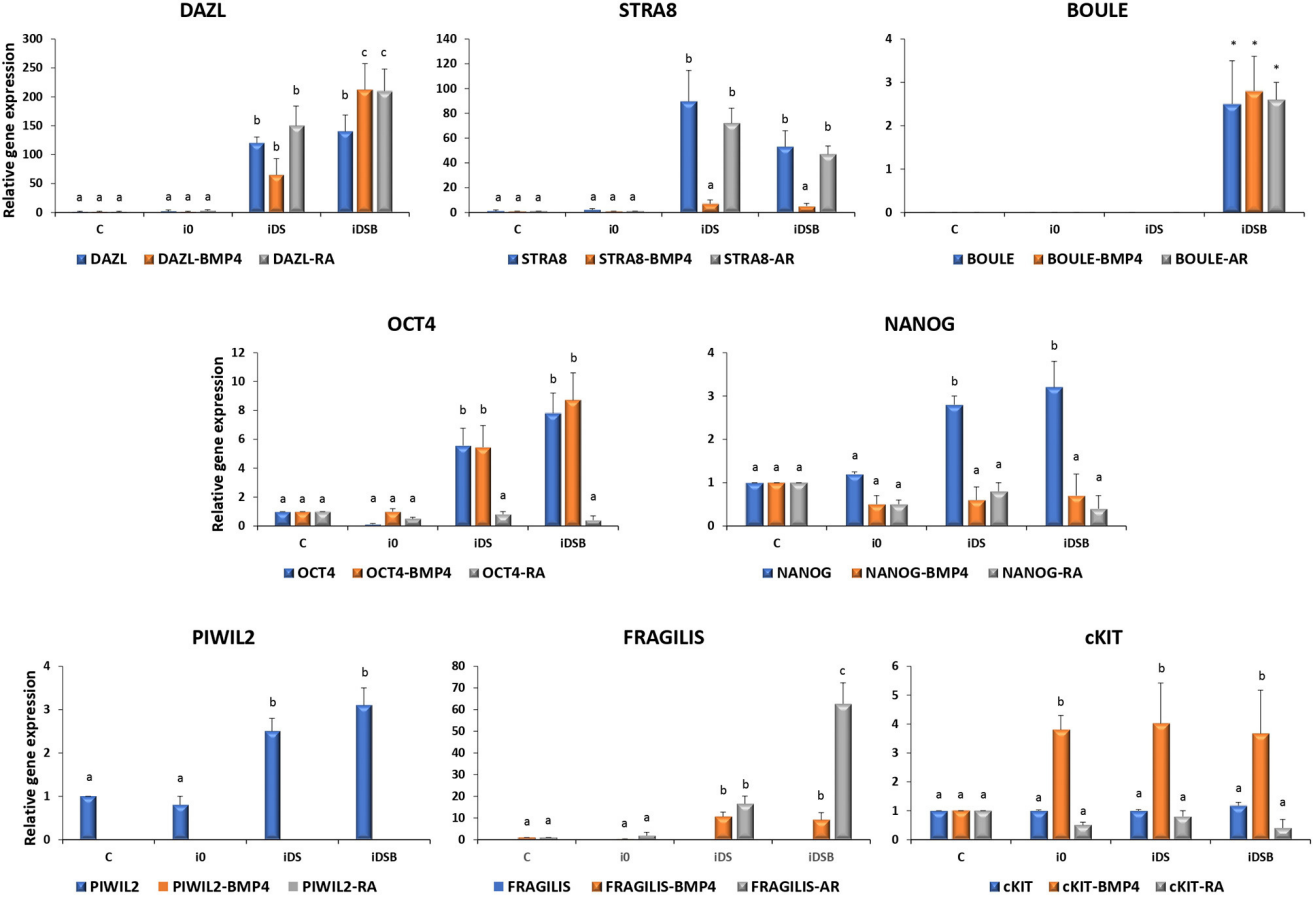
Gene expression analysis of pluripotency and GC-specific markers in bovine fetal AT-MSCs transfected with polycistronic vectors carrying combinations of DAZL, STRA8, and BOULE genes and treated with BMP4 or RA. Cells transfected with iDS or iDSB overexpressed (*P* < 0.05) DAZL and STRA8. Moreover, transfection of these plasmids in AT-MSCs induced the BOULE gene expression. Consequently, OCT4, NANOG, and PIWIL2 mRNA levels were upregulated (*P* < 0.05) in AT-MSCs transfected with iDS or iDSB. AT-MSCs transfected with iDS or iDSB and treated with 100 ng/mL of BMP4 for 14 days, increased (*P* < 0.05) the mRNA levels of FRAGILIS and cKIT compared to the BMP-untreated AT-MSCs. Transfection with iDS and treatment with 0.1 μM of RA for 14 days reduced OCT4, NANOG, and PIWIL2 but increased the FRAGILIS mRNA levels in AT-MSCs compared to the RA-untreated cells. Superscripts (a, b, c) indicate the significant differences (*P* < 0.05) for gene expression levels between transfection treatments.

Cells transfected with iDS or iDSB overexpressed (P < 0.05) DAZL (120.3- and 140.1-fold, respectively) and STRA8 (89.6- and 53.1-fold, respectively) Figure 3. Moreover, transfection of iDSB plasmid in AT-MSCs induced the BOULE gene expression (1-fold). Consequently, the OCT4 and NANOG mRNA levels were upregulated (*P* < 0.05) in the AT-MSCs transfected with iDS (5.6- and 2.8-fold, respectively) or iDSB (7.8- and 3.2-fold, respectively). Moreover, these cells increased (*P* < 0.05) the PIWIL2 mRNA levels when transfected with iDS or iDSB (2.5- and 3.1-fold, respectively).

AT-MSCs, transfected with iDS and treated with 100 ng/mL of BMP4 for 14 days, had similar (*P* < 0.05) mRNA levels of DAZL (65.1-fold), BOULE (1.3-fold), and OCT4 (5.5-fold) compared to the BMP4-untreated cells. However, this treatment increased (*P* < 0.05) the mRNA levels of FRAGILIS (10.6-fold) and cKIT (4-fold) and reduced (*P* < 0.05) the expression of STRA8 (7.1-fold), NANOG (0.6-fold), and PIWIL2 (undetected) in AT-MSCs compared to the BMP4-untreated cells. In comparison, AT-MSCs, transfected with iDSB and treated with BMP4 for 14 days, increased (*P* < 0.05) the expression of DAZL (212.3-fold), FRAGILIS (9.1-fold), and cKIT (3.7-fold) compared to the BMP4-untreated controls. Moreover, cells transfected with iDSB and treated with BMP4 had similar (*P* < 0.05) mRNA levels of BOULE (2.8-fold) and OCT4 (8.7-fold) but lower (*P* < 0.05) mRNA levels of STRA8 (5.2-fold), NANOG (0.7-fold), and PIWIL2 (undetected) compared to the BMP-untreated AT-MSCs.

Transfection with iDS and treatment with 0.1 μM of RA for 14 days resulted in similar mRNA levels of DAZL (150.1-fold), STRA8 (72.1-fold), BOULE (1.2-fold), and cKIT (0.8-fold) compared to the RA-untreated cells. Moreover, this treatment reduced OCT4 (0.8-fold), NANOG (0.8-fold), and PIWIL2 (undetected) but increased the FRAGILIS (16.6-fold) mRNA levels in AT-MSCs compared to the RA-untreated cells. AT-MSCs transfected with iDSB and exposed to RA for 14 days increased (*P* < 0.05) DAZL (210-fold) and FRAGILIS (62.7-fold) and had similar (*P* > 0.05) levels of STRA8 (47.5-fold), BOULE (2.6-fold), and cKIT (0.4-fold) mRNA levels compared to the RA-untreated cells. However, this treatment reduced (*P* < 0.05) the OCT4 (0.4-fold), NANOG (0.4-fold), and PIWIL2 (undetected) mRNA levels in AT-MSCs compared to the RA-untreated controls.

**Figure 4:**
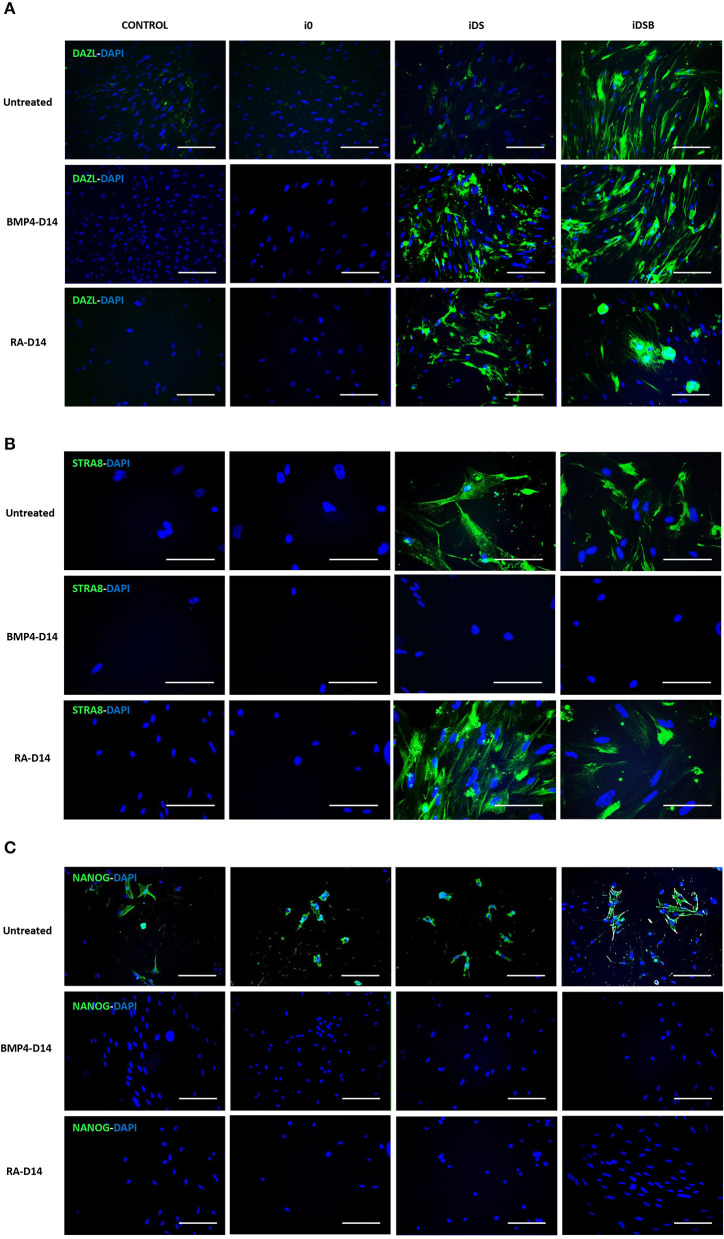
Immunolocalization of DAZL, STRA8, and NANOG in bovine fetal AT-MSCs transfected with polycistronic vectors carrying combinations of DAZL, STRA8, and BOULE genes and treated with BMP4 or RA. **(A)** Intense immunofluorescence associated with DAZL were observed in AT-MSCs transfected with iDSB. After treatment with BMP4 or RA for 14 days, immunoreactivity associated to DAZL remained in AT-MSCs transfected with iDSB. **(B)** STRA8 was immunolocalized in AT-MSCs transfected with iDS and iDSB and after treatment with RA. **(C)** Immunosignal associated with NANOG was observed in control cells and transfected AT-MSCs, however, BMP4 and RA treatments suppressed the NANOG immunosignal after 14 days of culture. Scale bars: 100 μm.

Intense immunofluorescence associated with DAZL, STRA8, and NANOG were observed in the AT-MSCs transfected with iDSB (Figures 4A,C). A weak DAZL immunosignal was also observed in the AT-MSCs transfected with iDS. In addition, STRA8 and NANOG were immunolocalized in the AT-MSCs transfected with iDS (Figures 4B,C). Immunosignal associated with NANOG was also observed in the control cells and AT-MSCs transfected with i0 (Figure 4C). After treatment with BMP4 for 14 days, immunoreactivity associated to DAZL remained in the AT-MSCs transfected with iDSB; however, this treatment resulted in the lack of STRA8 and NANOG immunosignal. Similarly, AT-MSCs transfected with iDS and treated with BMP4 were not immunoreactive to STRA8 and NANOG. Furthermore, after treatment with RA for 14 days, immunoreactivity to DAZL and STRA8 was observed in the AT-MSCs transfected either with iDSB or iDS. However, treatment with RA suppressed the NANOG immunoreactivity in these cells.

## Discussion

The gene expression patterns and tissue-specific localization of selected GC markers were analyzed in the bovine testis during the late fetal and adult stages. We found that the gene expression levels of OCT4, NANOG, c-KIT, DAZL, STRA8, BOULE, and PIWIL2 increased remarkably in the bovine testis from the fetal to adult stage. It has been reported that the OCT4 and NANOG expression expands from gonocytes in the neonatal testis to spermatocytes and round spermatids after sexual maturation, which may explain the increased expression levels found in the adult stage (25, 26). In concordance with these results, we immunodetected NANOG across the spermatogenic epithelium of the fetal testis with a high intensity in the Sertoli and pre-spermatogonial cells. Even though the roles of FRAGILIS and c-KIT have not been deeply studied in cattle, the expression of both genes occurs early in development, where they activate important mediators of postnatal meiotic and post-meiotic GC (26). In addition to the increasing expression levels of DAZL in the bovine testis during development, our analyses immunodetected DAZL mainly in the peripheral areas of bovine fetal seminiferous tubules associated to Sertoli and pre-spermatogonial cells. Similarly, DAZL gene expression levels have been shown to increase from pubertal to sexually mature sheep and dog testis, where it has been located in spermatogonia, primary and secondary spermatocytes (22, 27, 28). Moreover, we observed an immune signal associated to STRA8 in the spotted areas across the germinal epithelium in seminiferous tubules, showing a higher intensity in the basal compartment. STRA8 expression levels have been reported to peak at 6 months of age in goat testis and has been associated to the onset of meiosis with the highest intensity levels in preleptotene/early leptotene spermatocytes (29, 30). Levels of the BOULE expression in goat has been reported to increase in the adult compared to the immature testis, which may be associated to an increasing number of BOULE positive spermatogonial cells at the adult stage (31). Furthermore, the PIWIL2 gene expression pattern was in concordance to what was previously described in ovine, where high expression levels have been detected in sexually mature gonadal tissues, mainly localized in spermatogonia, primary spermatocyte, and oocyte (32). Thus, the overall increased expression levels of DAZL, STRA8, and BOULE in the adult testis and cell-specific distribution of DAZL and STRA8 in the fetal testis suggest that these genes are important components of the regulatory network that control the *in vivo* differentiation of bovine GCs.

In order to mimic the gene-regulatory complex that activates the GC development *in vivo*, we then sought to develop a multigene overexpression system using polycistronic vectors carrying combinations of DAZL, STRA8, and BOULE to induce GC differentiation of bovine fetal AT-MSCs. Polycistronic constructs have been successfully used for the co-expression of genes in a wide variety of biotechnological applications including iPSC production and gene therapy (33–35). In the current study, transcription of polycistronic vectors in bovine fetal AT-MSCs induced a positional expression effect in second position gene STRA8 in tri-cistronic vectors. Tri-cistronic constructs induce a higher expression in first positions genes compared to second and third position sequences, which may explain why it was lower in the STRA8 expression. Moreover, a gradual decrease in the transcribed mRNA along the vector due to the naturally occurring ribosome drop-off has been described in long sequence transcripts (36). Despite the increased transcript length, BOULE gene located in the third position in tri-cistrionic vector increased its expression compared to the controls.

Nevertheless, in the present study, overexpression of DAZL and STRA8 in bi-cistronic and DAZL, STRA8, and BOULE in tri-cistronic vectors resulted in the upregulation of OCT4, NANOG, and PIWIL2 in bovine fetal AT-MSCs. During spermatogenesis, DAZL expression overlaps with OCT4 and NANOG, until OCT4 is downregulated when cells enter meiosis at the type B spermatogonia stage and NANOG levels decrease when migrating PGCs reach the gonad (37). Previously, it has been reported that a combined or individual overexpression of DAZL, STRA8, or BOULE in goat BM-MSCs resulted in the upregulation of OCT4 (22, 23). Even though the molecular mechanism of STRA8 remains unclear, recently, it has been reported that STRA8 may regulate spermatogenesis *via* the Cdl4-Clu4A-Ddb1 ubiquitinated degradation axis in a proliferating cell nuclear antigen (PCNA)-dependent manner (38). On the other hand, DAZL has been associated to several molecules including the polyribosomes and poly(A)-binding proteins (PABPs), which suggests its participation in several aspects of protein synthesis including mRNA stability, transportation, localization, or translation (39). Despite the reported concomitant activities of DAZL and BOULE in spermatogenesis (21), our data showed no synergistic effect after BOULE inclusion in tri-cistronic vectors. Although the mechanism whereby DAZL or STRA8 regulates the level of OCT4 and NANOG is currently unclear, our data suggest that STRA8 and DAZL may regulate pluripotency through the activation of these transcription factors.

Treatment with BMP4 increased the expression of DAZL in bovine fetal AT-MSCs transfected with tri-cistrionic vector. We have previously reported that the un-transfected bovine fetal AT-MSCs treated with the same concentration of BMP4 increased the DAZL mRNA levels at Day 14 of culture (7). BMP4 activity is mediated by receptor ALK3 and transducer SMAD5, exerting both mitogenic and differentiative effects (40). In the present study, while BMP4 repressed the NANOG expression, this treatment also activated FRAGILIS and increased the c-KIT gene expression levels in bovine fetal AT-MSCs. BMP4 control over the FRAGILIS expression has been widely studied in mice development, mediated through BMP4 signaling secreted from the extra-embryonic ectoderm (12). Evidence sustaining the BMP4-activation of FRAGILIS is supported by studies showing a lack of the FRAGILIS positive cells in BMP4-null embryos and significant reduction in BMP4 heterozygous embryos (12). Moreover, c-KIT is also be activated by BMP4 in spermatogonial cells, *via* Alk3 and the R-Smad Smad5 stimulation, resulting in both mitogenic and differentiative effects (40). Overall, these data suggest that BMP4 effect may control differentiation of bovine fetal AT-MSCs by reducing pluripotency and activating gene expression of early GC markers including DAZL, FRAGILIS, and c-KIT.

Treatment with RA for 14 days increased the expression of DAZL and FRAGILIS, and maintained the mRNA levels of STRA8 in bovine fetal AT-MSCs transfected with bi-cistronic and tri-cistronic vectors. Moreover, RA treatment repressed the expression of OCT4 and NANOG in these cells. Our previous analyses in bovine fetal BM-MSC showed that the NANOG levels were not affected by RA; however, this treatment increased the expression of DAZL and reduced the expression of OCT4 (7). RA is a multipurpose factor that controls the meiotic entrance of PGCs in the genital cords by controlling the Cyp26b1 enzyme expression (15). RA activity has also been reported in the ESCs resulting in the increased gene expression levels of several GC genes including FRAGILIS and STRA8 (41). Activity of RA is exerted inside the nucleus during gametogenesis through their binding to the RA receptors (RAR) in several cell types including Sertoli cells (RARα), round spermatids (RARβ), and type A spermatogonia (RARγ) (42). Even though the signaling pathway of RA has not been entirely described in MSCs, these results suggest that RA partially activates the GC gene expression pattern by upregulating DAZL and STRA8 in overexpressing AT-MSCs. Recently, it has been reported that the BMP4 and RA signaling pathways play opposing roles in GC formation (43). BMP4 regulates PGC formation through histone acetylation and DNA methylation in the DAZL gene. In comparison, RA binds RARα which is regulated by STRA8 promoter activity during SSC formation (43).

In conclusion, gene expression and immunohistochemical data suggest that DAZL, STRA8, and BOULE are important components of the regulatory network that control the *in vivo* differentiation of bovine GCs. Overexpression of DAZL, STRA8, and BOULE induced the upregulation of pluripotency markers and PIWIL2 in the transfected bovine fetal AT-MSCs. BMP4 and RA partial activation of the GC gene expression suggests that both factors possess common targets but induce different gene expression effects during the GC differentiation in overexpressing bovine fetal AT-MSCs.

## Data Availability Statement

The raw data supporting the conclusions of this article will be made available by the authors, without undue reservation.

## Ethical Statement

The animal study was reviewed and approved by Bioethical Committee of the National Commission for Scientific and Technology Research from Chile (Fondecyt).

## Author Contributions

MDR, CGT, MV-G, VHP, and OAP contributed to the conception and design of the study, acquisition of data, analysis, and interpretation of data, drafting the article, writing, review and editing, and revising the article critically for important intellectual content. PC, AG-M, and JC performed the experiments and analyzed the data. All authors contributed to the article and approved the submitted version.

## Conflict of Interest

The authors declare that the research was conducted in the absence of any commercial or financial relationships that could be construed as a potential conflict of interest.
